# Impact of group work on the hidden curriculum that induces students’ unprofessional behavior toward faculty

**DOI:** 10.1186/s12909-024-05713-7

**Published:** 2024-07-19

**Authors:** Aoba Nakamura, Hajime Kasai, Mayumi Asahina, Yu Kamata, Kiyoshi Shikino, Ikuo Shimizu, Misaki Onodera, Yasuhiko Kimura, Hiroshi Tajima, Kazuyo Yamauchi, Shoichi Ito

**Affiliations:** 1 Department of Medicine, School of Medicine, Chiba University, Chiba, Japan; 2 Department of Medical Education, Graduate School of Medicine, Chiba University, Chiba, Japan; 3 Health Professional Development Centre, Chiba University Hospital, Chiba, Japan; 4 Department of Respirorolgy, Graduate School of Medicine, Chiba University, Chiba, Japan; 5 Department of Community-oriented Medical Education, Graduate School of Medicine, Chiba University, Chiba, Japan

**Keywords:** Professionalism, Unprofessional behavior, Hidden curriculum, Affinity diagram

## Abstract

**Background:**

Hidden curriculum (HC) can limit the effects of professionalism education. However, the research on how HC triggers unprofessional behavior among medical students is scant. Furthermore, there is no established approach for how faculty members may create a context, such as an educational environment and education system, that prevents students’ unprofessional behavior. This study aimed to develop an educational approach to prevent unprofessional behavior and clarify how faculty members consider HC that triggers students’ unprofessional behavior.

**Methods:**

The study sample comprised 44 faculty members and eight medical students from the Chiba University School of Medicine. The participants were divided into groups and asked the following question: “What attitudes, statements, and behaviors of senior students, physicians, and faculty members trigger medical students’ unprofessional behavior?” The responses were collected using the affinity diagram method. The group members discussed the causes and countermeasures for the selected attitudes, statements, and behaviors of senior students, physicians, and faculty members based on the affinity diagram. The impact of the group work on the faculty members was surveyed using questionnaires immediately after its completion and six months later. Furthermore, the cards in the group work were analyzed using content analysis.

**Results:**

The responses to the questionnaire on group work indicated that some faculty members (43.8%) improved HC, while others suggested conducting group work with more participants. The content analysis revealed six categories – inappropriate attitude/behavior, behavior encouraging unprofessional behavior, lack of compliance with regulations, harassment of other medical staff, inappropriate educational environment/supervisor, and inappropriate self-control – and 46 subcategories.

**Conclusions:**

The HC that triggers students’ unprofessional behavior includes the words and actions of the educator, organizational culture, and educational environment. Group work makes faculty members aware of the HC that triggers unprofessional behavior, and induces behavioral change for HC improvement in the educational activities. Educators should refrain from using words and actions that encourage unprofessional behavior, such as personal anecdotes, as they reduce students’ learning motivation.

**Supplementary Information:**

The online version contains supplementary material available at 10.1186/s12909-024-05713-7.

## Background

Professionalism is both a quality and an ability for physicians. Although professionalism can be influenced by time, culture, and geography [1], the definition proposed by Arnold and Stern is widely accepted [2]. It presented that professionalism consists of excellence, humanity, accountability, and altruism on a foundation of clinical competence, communication skills, and ethical and legal understanding [2]. It is presented in the Medial Professionalism in the New Millennium: A Physician Charter [3]. Professionalism is a competence that must be acquired and practiced, and professionalism education is provided in medical school. However, problematic, unprofessional behavior among medical students and physicians is increasing, with patients’ and society’s trust in medical students and physicians fluctuating accordingly. One notable problem is the inappropriate – “unprofessional” – behavior of physicians [4]. Professionalism education has two goals. First is the foundational objective of instructing students to practice medical professionalism, thereby, discouraging engagement in unprofessional behavior. Second is the aspirational objective, entailing a commitment to consistently aim high and strive to become an exemplary medical professional [5]. Examples of unprofessional behavior include not being involved in classes and other activities, dishonest or disrespectful behavior, and low self-awareness [6]. In addition, medical students who exhibit unprofessional behavior during medical school are at a significantly high risk of receiving disciplinary action after graduation [7], and faculty members are expected to recognize and manage students’ unprofessional behavior [6].

The effectiveness of professionalism education can be limited by situations outside the formal curriculum, such as classes and extracurricular activities; referred to as the “hidden curriculum” (HC) [8, 9]. Medical students can be influenced by the attitudes and behaviors of senior students, physicians, and faculty members [10]. HC arises from students’ observations of healthcare providers’ behavior, speech, tone, attitudes toward patients and the environment, and professional life [11, 12]. Medical students and residents observe unethical and unprofessional behaviors among colleagues and supervisors [13, 14]. Furthermore, such inappropriate HC triggers unprofessional behavior, which conflicts with professionalism education.

Professionalism education is practiced throughout Asia [15–19]. Professionalism in the Asian context has certain similarities and differences with the West [1]. Medical professionalism attributes in non-Western cultures are influenced by cultural dimensions and values [1, 20]. There have been several reports of unprofessional behavior in Asian countries [21–24]. However, since there have been limited reports about HC in Asia, including Japan [25–27], it is unclear whether HC throughout Asia has similar characteristics in terms of unprofessional behavior.

Faculty development (FD) and reflection may serve as important interventions for managing HC [9, 28, 29]. However, few studies have examined the appropriate approaches for students and faculty members to address HC that may cause medical students’ unprofessional behavior [11, 30]. The concepts and elements of HC have been evaluated in interviews with only students [10, 25, 31], with students and faculty members [32], and in scoping reviews [33]. However, it remains unclear what faculty members may consider to be HC that triggers students’ unprofessional behavior. There is no established approach by which faculty members can create an educational environment that discourages unprofessional student behavior. There are differences in faculty members’ and medical students’ perceptions of professional and unprofessional behaviors [34]. Hence, implementing an approach for curbing HC that faculty members consider appropriate may not be effective due to the different perceptions.

To address these knowledge gaps, this study aimed to elucidate the factors that faculty members may perceive as contributing to students’ unprofessional behavior. In addition, the study seeks to develop an educational approach that enables faculty members to proactively mitigate instances of such behavior. Although it is difficult to be completely explicit about HC, open discussions between educators and students can facilitate its exploration [10]. Thus, this study hypothesizes that group work focused on HC and involving both faculty members and students will enhance faculty awareness of HC. It further hypothesizes that group work will influence their behavior toward HC.

## Methods

### Ethical approval

This study was approved by the Ethics Committee of Chiba University (approval no. 3425). The study database was anonymized.

### Study design

**Fig. 1:**
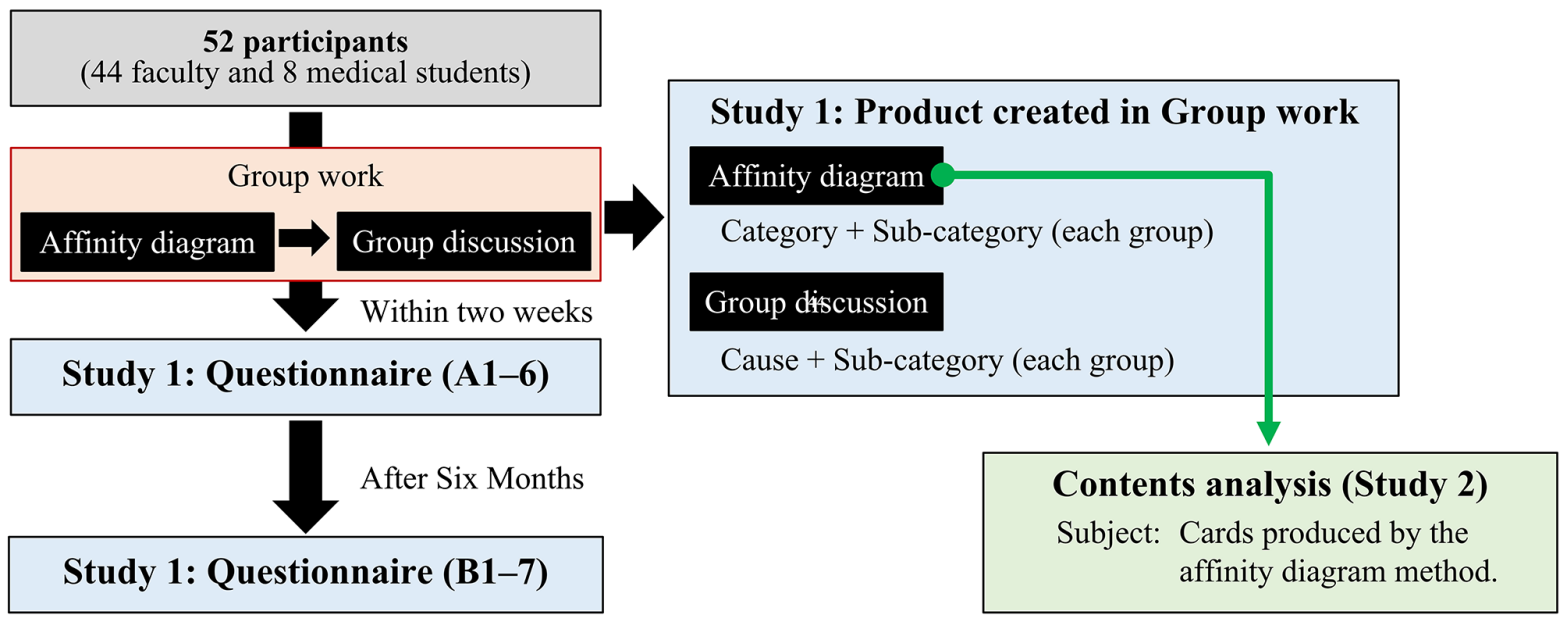
Flow diagram of the design

This study had an explanatory sequential mixed methods design (Fig. 1). In the first part (Study 1), the group work’s effectiveness with the affinity diagram method and HC group discussion was studied using a combination of quantitative data and qualitative analysis. In the second part of the qualitative analysis (Study 2), a content analysis was conducted for the cards in the group work to identify HC categories to provide suggestions for responding to each category.

### Setting

#### Participants

The group work took place in August 2022 during the annual intramural conference on medical education held at Chiba University. Faculty members in the basic medicine and clinical medicine departments and medical students at the Chiba University School of Medicine participated.

One faculty member was nominated from each department. They were selected either based on their own request or the recommendation of their respective departments. The faculty members did not specify any conditions for the designation of participants other than their job title – professor, associate professor, and assistant professor practicing medical education. One to two medical students were invited from each year (years 1 to 6). The students were designated as representatives at the start of the university to contact and coordinate with their respective years. The representatives for each year were selected by the students. No exclusion criteria were established.

In qualitative research, there are no clear criteria for sample size, which can be determined by a saturation of opinions [35]. As this study was exploratory, incorporating both quantitative and qualitative research through the collection of opinions in group work, no a priori settings regarding sample size were established. Thus, the sample size was a feasible number depending on the number of departments for the faculty members and the number of medical students from each year.

##### Informed consent

Informed consent to use the products of the group work was obtained from the participants before they engaged in the group work. Additionally, informed consent was obtained through the explanations included in the survey form provided to the participants. The participants were divided into groups of 6 to 8 participants, consisting of both faculty members and medical students. The ratio of faculty members to students and faculty members’ areas of expertise and job titles remained unbiased.

#### Group work with the affinity diagram method

In the group work, responsible members of the Department of Medical Education (HK) provided specific examples of students’ unprofessional behavior using a previous report and explained the definition of HC [6].

**Fig. 2:**
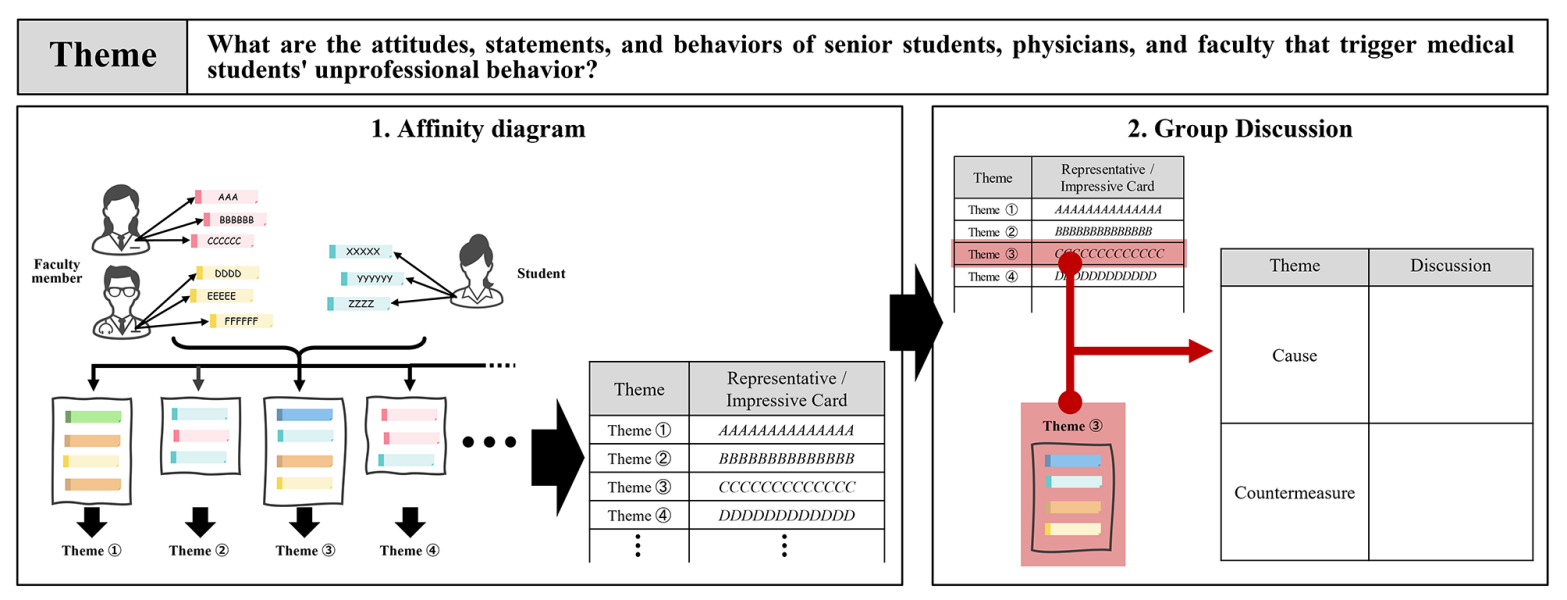
The process of group work in our study

Thereafter, the participants were asked the following question: “What attitudes, statements, and behaviors of senior students, physicians, and faculty members trigger medical students’ unprofessional behavior?” The affinity diagram and group discussion methods were explained. The affinity diagram (Fig. 2) compiled the qualitative data and constructed a new meaning system [36, 37]. This method was chosen because it allowed the faculty members and students to brainstorm and formulate their opinions in a limited timeframe. The affinity diagram in this study was constructed as follows:

1. Members of each group spread out a large sheet of paper on their desks.
2. Each member wrote one opinion on the theme on a card (Post-it^®^) and placed the card on the paper. During this time, the group members did not exchange opinions.
3. When each member had written at least five cards, all the team members looked over the cards placed on the paper.
4. The cards with similar contents were grouped into one category.
5. A theme that expressed the common elements of the categories was prepared; the summary was used as the name of each category.

The themes created in each group were entered into a table provided for each group. The opinions in the affinity diagram were not restricted to participants’ actual experiences.

Each group selected one of the themes based on the cards collected using the affinity diagram. The group members discussed the causes and countermeasures for the selected attitudes, statements, and behaviors of senior students, physicians, and faculty members. The results of the group discussions were summarized in a table. All the participants gathered and shared their opinions with each group. The results and the themes were summarized in the tables provided for each group.

This group work was conducted for 90 min–10 min for orientation, 50 min for affinity diagram and group discussion, and 30 min for overall information sharing.

### Data collection and analysis

#### Group work products

The categories in the affinity diagram created by each group, the theme selected by each group for group discussion, and the group discussion on causes and countermeasures were collected based on the tables created by each group. Furthermore, the common themes were categorized as one theme, with the types of themes as subcategories.

#### Questionnaire

An anonymous questionnaire was administered to evaluate the effects of the group work on participants’ responses to HC. After the group work, the participants responded to the following questionnaire items (S. Table 1A): (A1) How useful was the group work?, (A2) How difficult was the group work?, and (A3) How actively did you participate in the group work? All the questions were scored on a 7-point Likert scale, ranging from 1 [(A1) Not useful at all, (A2) Very easy, and (A3) Very negative] to 7 [(A1) Very useful, (A2) Very difficult, and (A3) Very positive]. The score of “4” was set to “Neither” for (A1) and (A3), and to “Appropriate difficulty” for (A2). Furthermore, they were asked the following questions (S. Table 1A): (A4) What new things did you learn from this group work?, (A5) What was a good part of this group work?, and (A6) What was the negative aspect of this group work?

In March 2023, six months after the group work was conducted, a questionnaire was sent to the participating faculty members to evaluate if a change in behavior regarding educational activities had occurred owing to their participation. As the survey targeted behavioral changes in faculty members toward HC, medical students were excluded from the survey. The participants responded to the following questionnaire items (S. Table 1B): (B1) To what extent did your daily educational behavior change as a result of the group work?, (B2) To what extent have you become more aware of other faculty members’ and physicians’ behaviors and environments that trigger students’ unprofessional behavior?, and (B3) Would you like to participate in such professionalism training again? All the questions were scored on a 7-point Likert scale, ranging from 1 [(B1) Not changed at all, (B2) Not careful at all, and (B3) Do not want to participate at all] to 7 [(B1) Very much changed, (B2) Very careful, and (B3) Want to participate very much]. The score of “4” was set to “Neither” for (B1)–(B3). In addition, they were asked the following questions to evaluate the long-term effects of the group work (S. Table 1B): (B4) What did you implement after the group work to educate students about professionalism?, (B5) What did you stop implementing for students’ professionalism education after the group work?, and (B6) Do you have any additional comments or questions regarding the group work?

The questionnaire was designed for program evaluation. A Likert scale was used as it was easy for the participants to answer. The questionnaire items were created under the supervision of physicians specializing in medical education (HK, MA, KS, and SI).

#### Study 1

*Quantitative data collection and analysis*. Quantitative data were collected using the questionnaire immediately after the group work and six months later (Questions (A1)–(A3) and Questions (B1)–(B3), respectively) to evaluate the effects of the group work on participants’ responses to HC. Quantitative data were expressed in terms of mean ± standard deviation (SD) unless otherwise indicated. All statistical analyses were performed using JMP 16.0 (Cary, NC, USA).

*Qualitative data collection and analysis.* Qualitative data were collected using questionnaires immediately after the group work and six months later (Questions (A4)–(A6) and Questions (B4)–(B6), respectively) to evaluate the effects of the group work on the participants’ responses to HC. As the number of responses to the open-ended items was small, the responses were categorized according to their content and representative opinions.

#### Study 2

*Qualitative data collection and analysis*. Each card created by the participants in the group work was considered as one opinion on the attitudes, statements, and behaviors of senior students, physicians, and faculty members that trigger medical students’ unprofessional behavior. Following previous studies, qualitative content analysis was performed to analyze the opinions formed during the group work [38]. The analysis comprised the descriptions of the manifested content and interpretations of latent content [39]. In field of professionalism education for healthcare professionals, content analysis of reflections and interviews after educational practice has been reported [40–42].

The content of the cards made by the participants during the group work was transcribed and listed. These opinions were coded and categorized according to the content analysis method. In the primary analysis, the authors (AN and HK) independently read all the opinions. They coded and categorized them via paraphrasing, such as abstraction, and formed subcategories. In the secondary analysis, the subcategories were classified into higher levels and integrated into categories. Inter-rater reliability was evaluated by the Kappa coefficient (0.8–1.0 = almost perfect; 0.6–0.8 = substantial; 0.4–0.6 = moderate; 0.2–0.4 = fair) [43]. After the codes were revised, the Kappa value was 0.71. In cases of disagreement over coding, the authors discussed the codes until a consensus was achieved. This process was supervised by the co-authors (MA, KS, IS, HT, KY, and SI) specializing in medical professionalism education.

## Results

The study included 52 participants – 44 faculty members (professors, *n* = 14; associate professors, *n* = 13; lecturers, *n* = 11; assistant professors; *n* = 5; and others, *n* = 1) and 8 medical students (first year, *n* = 2; second year, *n* = 1; third year, *n* = 2; fifth year, *n* = 2; and sixth year, *n* = 1) from the Chiba University School of Medicine. The participants were divided into seven groups, each of which engaged in group work and discussions.

### Study 1

#### Group work results

The affinity diagram method resulted in 3–6 themes for each group. Each group discussed the themes to determine the causes and countermeasures of HC – assessment, harassment, ethics, physicians’ statements and attitudes, personal anecdotes, inappropriate attitudes as a medical professional, and students’ reduced learning motivation. For the HC selected for discussion in each group, 2–6 causes, such as generation gap, hierarchical context, lack of respect for patients, other doctors, and health professionals, lack of appropriate teaching techniques, and insufficient time and effort for education, were listed. Each group suggested 3–7 countermeasures, such as FD for HC, students’ feedback on HC to faculty members, information sharing between students and faculty members and between faculty members and physicians, and a review of the working environment.

Table 1 presents the worksheets of the two groups. Both groups identified five themes. The members in Groups 1 and 2 focused on “assessment” and “physicians’ statements and attitudes,” respectively. Group 1’s members discussed the causes to be a large number of students, insufficient time for education, no attending physician, no working relationship between students and faculty members, and the difficulty of providing feedback. As a countermeasure, they suggested constructing a model for teaching methods, unifying patterns of education, and providing frequent feedback. Group 2’s members discussed the causes to be the distinction made by supervising physicians based on students’ aspirations, the behaviors and attitudes of physicians that drive students to tolerate such behaviors, generational gap, misplaced sense of time, and excessive self-consciousness. They suggested opportunities for educators to increase their HC awareness as a countermeasure.

**Table 1:** Examples of the themes created by each group using the affinity diagram method

Group	Themes of category	Selected theme	Cause	Management
1	1. Attitude of medical personnel 2. Manners 3. Hierarchy 4. Assessment 5. Selfish	· Assessment	· Too many students relative to supervisors · Not enough time for education · No attending physician, or if there are, they are not functional. · No working relationship between students and faculty · Difficulty in how faculty gives feedback during clinical clerkship	· Education center offers a model for teaching methods · Unify patterns of education · Frequent feedback
2	1. Physicians’ statements and attitudes 2. Clothing and appearance 3. Morals 4. Responses during teaching 5. Teamwork	· Physicians’ statements and attitudes	· Modifying approaches toward individual students based on potential future relationships · Engaging in gossip about patient evaluations and information, leading students to perceive it as acceptable behavior	· Creating opportunities for all educators to be aware of the impact of their statements · Providing chances for educators to reflect on their responses in education, similar to this group work opportunity · Establishing systems for receiving feedback from students · Fostering an environment where opinions can be freely expressed, even to leaders and senior staff within the organization

**Fig. 3:**
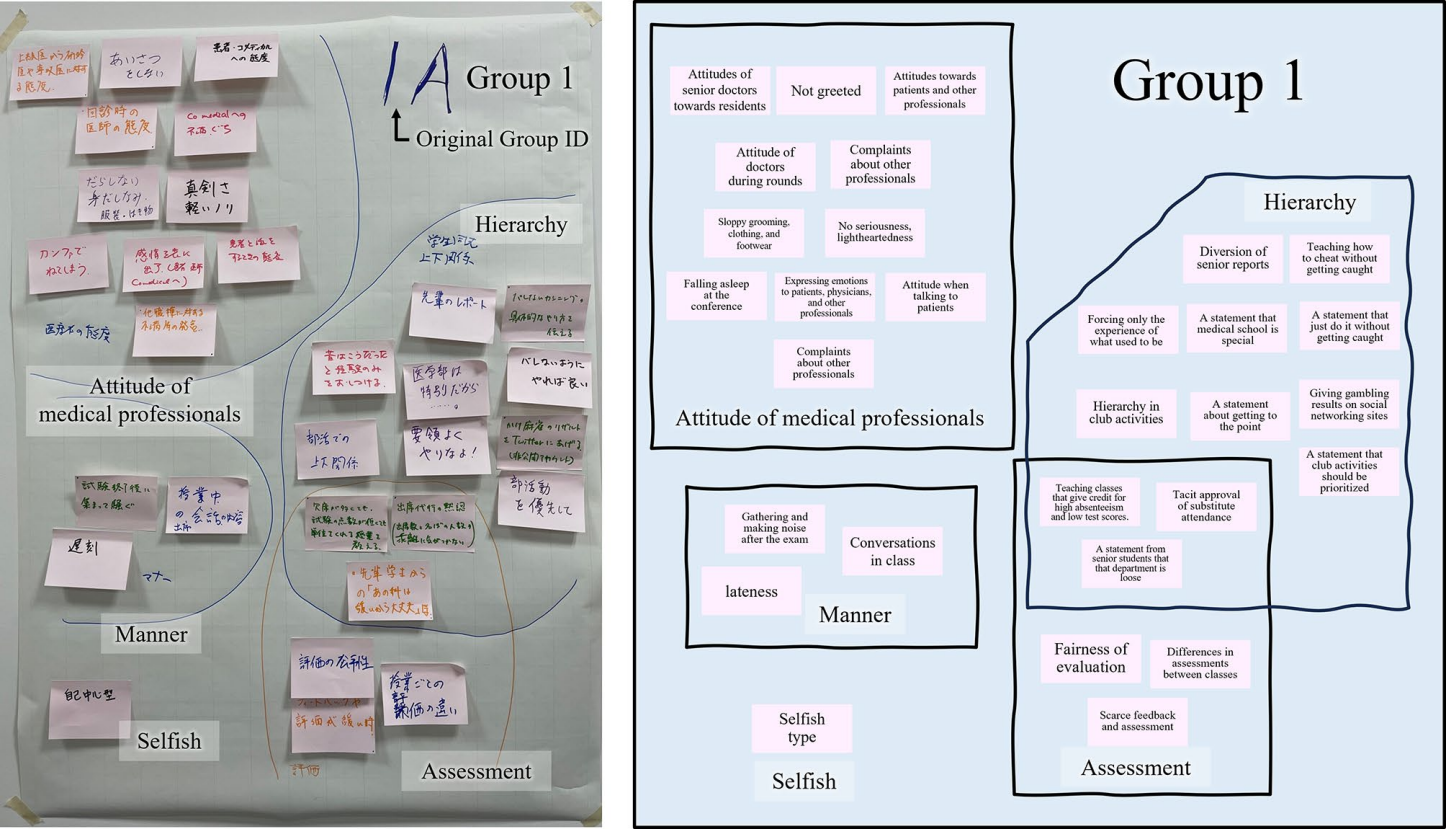
Product made by Group 1. The product was made using the affinity diagram method and schema that translates the product into English

Figure 3 shows the affinity diagram products of Group 1. The results of the other groups are summarized in S. Table 2.

**Table 2:** Representative comments from participants on A4–A6 and B4–B6.

Items	Comments
Questionnaire after the group work	
(A4) What new things did you learn from this group work?	“I learned that the students are carefully observing what the faculty members say and do.”
	“I noticed that my actions as a supervisor have a great deal to do with education.”
(A5) What was a positive aspect of this group work?	“It was very refreshing and informative to have the students participate in the group discussion. It was a good opportunity to hear students’ opinions and review ourselves.”
(A6) What was the negative aspect of this group work?	“Those who participate in the group work do not necessarily have a sense of responsibility to communicate and teach their course’s faculty members and graduate students …. Therefore, only those who have participated may be left with the awareness and impact of this event.”
Questionnaire six months after the group work	
(B4) What did you implement after the group work to educate students about professionalism?	“I began to advise other faculty members on dress code, statements, and actions.
	“I became aware that I was always being observed by the students.”
(B5) What did you stop implementing for students’ professionalism education after the group work?	“I said members of my department should refrain from making comments that are not related to their practice during the conference.”
	“Not to rescue students who had failed the re-examination.”
	“Talking about my student days has become less frequent.”
(B6) Do you have any additional comments or questions regarding the group work?	“I think it should be implemented by all means with a larger number of participants.”
	“I think it would be good to continue this group work and expand the range of participants.”

#### Questionnaire results

The questionnaire was provided immediately after 38 of the 52 participants (63.3%) completed the group work. The responses indicated that the group work was useful and acceptable for the participants ((A1) usefulness = 6.0 ± 1.1; (A2) difficulty = 4.2 ± 0.6, and (A3) positive attitude toward group work = 5.5 ± 1.0).

Comments on A4 (New things learned from the group work) were noted by 11 (28.9%) out of 38 participants. These comments were divided into the following categoriesthe opportunity to reconsider education (*n* = 3), the importance of exchanging opinions on education (*n* = 3), the concept of HC (*n* = 2), the concept of role models (*n* = 2), and the concept of unprofessional behavior (*n* = 1). Comments on A5 (What was a A good part of the group work) were noted by 11 participants (28.9%). These comments were divided into discussions between students and teachers (*n* = 7), discussions between teachers from different departments (*n* = 3), and encouragement of their awareness (*n* = 1). Comments on A6 (The negative aspect of the group work) were noted by four participants (10.5%). These comments were divided into the limited number of participants (*n* = 3) and insufficient time for group work (*n* = 1). Representative comments from participants on A4–A6 are summarized in Table 2.

The questionnaire was again administered after six months after 16 of the 44 participants (30.7%) completed the group work. The responses indicated that the group work encouraged changes in the educational behavior of the participants ((B1) changes in educational activities = 4.6 ± 1.3 and (B2) changes in response to other faculty members’ and physicians’ unprofessional behavior = 5.3 ± 1.1). In addition, the participants were highly motivated to participate in the group work again ((B3) = 5.1 ± 1.5).

Based on the responses to B4–B6, seven of the 13 faculty members (43.8%) made improvements in HC. In addition, three faculty members (18.8%) suggested that this group work should be conducted with a wider range of participants. Representative comments from participants on B4–B6 are summarized in Table 2.

### Study 2

#### Content analysis

The affinity diagram method yielded 241 cards of which 237 cards were analyzed as valid data. Those that were inconsistent with the theme of this study or were difficult to read were excluded. As a result, the cards were classified into six categories – inappropriate attitude/behavior, behavior encouraging unprofessional behavior, lack of compliance with regulations, harassment of other medical staff, inappropriate educational environment/supervisor, and inappropriate self-control – with 46 subcategories (Table 3).

**Table 3:** Absolute frequencies of codes for each category

Category	Subcategory	*n*	Quotes
Inappropriate attitude/behavior	Lack of respect for patients	26	*Arrogant attitude toward patients.* (F5-3)
(*n* = 77)	Lack of respect for colleagues and other medical professions	20	*Making negative comments about others’ statements behind their backs.* (F107-1)
	Inappropriate statements and behavior	12	*Unsuitable remarks in public situations (e.g., conferences, etc.)* (F108-1)
	Lack of integrity	6	*Light-hearted flirtation without seriousness.* (F2-2)
	Lack of communication	4	*Not discussing the issue adequately because they are ‘busy’.* (F16-1)
	Dozing	3	*Falling asleep at the conference.* (F3-2)
	Lack of etiquette	2	*Faculty (senior staff) do not return greetings.* (F115-1)
	Lack of cooperation	2	*Denial of teamwork.* (F94-1)
	Inadequate medical record entries	1	*Incomplete medical record.* (F14-3)
	Rejection of feedback to self	1	*Avoiding criticism and other feedback on one’s behavior.* (F66-1)
	Egoism	1	*Self-centered thinking.* (F2-5)
Statements recommending unprofessional behavior	Encouragement/affirmation of not trying	42	*A senior colleague makes fun of the seriousness with which you approach your practical training*. (F99-1)
(*n* = 64)	Encouraging cheating	10	*Teach (specific) ways to cheat without getting caught.* (S2-2)
	Traditions of the past	10	*Talking about inappropriate behavior during school days in a saga-like manner*. (F56-4)
	False sense of privilege	1	*The statement, ‘Medical school is special, so it’s okay’.* (F1-3)
	No alerts for each other	1	*Lack of restraint within the student group*. (S5-2)
Lack of compliance with regulations	Poor personal appearance	13	*Wearing a white coat sloppily.* (F81-1)
(*n* = 49)	Failure to be punctual	11	*Class overtime*. (F12-4)
	Illegal behavior	6	*Forcing a minor to drink alcohol*. (F38-1)
	Inappropriate use of social networking sites (SNS)	5	*Arguments (accusations, slander) on SNS*. (F5-2)
	Cheating	5	*Fabrication of report data*. (F68-1)
	Abuse of confidentiality	4	*Conversations regarding personal information not related to the medical examination*. (F76-3)
	Unauthorized absence	3	*Non-participation in conferences*. (F59-1)
	Forgetting something	1	*Forgetting something*. (F12-2)
	Failure to follow infection precautions	1	*Failure to follow infection precautions*. (F6-2)
Harassment of other medical personnel	Abuse of authority	26	*The physician has a coercive attitude toward patients and other healthcare professionals*. (S7-3)
(*n* = 35)	Sexual harassment	4	*Sexually harassing statements and behavior*. (F9-4)
	Discrimination	4	*Change attitudes toward patients and health care professionals based on likes and dislikes.* (F30-1)
	Wrong hierarchical relationship	1	*Hierarchy in club activities*. (F4-3)
Inappropriate educational environment and leadership	Indifference to the learner	7	*Not paying attention to what you do in class*. (F22-1)
(*n* = 31)	Inappropriate learner evaluation	6	*Differences in evaluations from class to class*. (F3-5)
	Unfair attitude	6	*The response changes depending on who you are dealing with.* (F33-1)
	Excessive burden requirements	2	*Overburdening students without considering their condition*. (F36-1)
	Lack of explanation	2	*They don’t explain by saying, ‘You wouldn’t understand this surgery anyway’.* (F73-1)
	Acceptance of cheating	2	*Tacit approval of substitute attendance*. (S2-5)
	Biased ideology	2	*Criticize or deny specific ideas*. (F17-5)
	The sudden cancellation of classes	1	*Sudden class cancellation*. (F12-5)
	old-fashioned values	1	*‘Can a student who leaves on time work as a doctor?’ said a doctor*. (F10-2)
	Heterogeneity of instruction	1	*Uneven sense of being a leader*. (F32-1)
	Teaching uncertain information	1	*Communicating uncertain information*. (F15-3)
Inappropriate self-management	Inappropriate drinking	2	*Medical treatment in a state of hangover*. (F56-3)
(*n* = 7)	Smoking	2	*Smoking in non-smoking areas*. (F48-1)
	Not taking rest	1	*Tired physicians at the hospital where they practice.* (F111-1)
	Lack of organization	1	*Reorganization of desks, etc. in physicians’ offices, etc.* (F76-5)

## Discussion

The group work in which faculty members and students discuss HC using the affinity diagram method can improve faculty members’ awareness of HC and drive behavioral changes to improve HC. Furthermore, senior students, physicians, faculty members, and the educational environment can act as HC that triggers students’ unprofessional behavior. In addition, comments that negate students’ learning efforts and convey the unprofessional behavior of senior students, physicians, and faculty members, such as personal anecdotes, can become HC for medical students.

Previous studies have examined HC management for unprofessional behavior [8, 11, 33]. A scoping review identified the key components of HC as the structure and rules of the medical education organization, the dominant culture of the educational environment, the teaching and assessment approach, and the physical, clinical, and educational settings [33]. Interpretive structural modeling has shown that role-modeling behaviors and interpersonal relationships (social factors) are influenced by underlying organizational and educational factors [33]. Chapa et al. conducted a useful program for students on HC management [8]. This study is the first to evaluate the effect on faculty members through group work on HC that triggers unprofessional behavior between faculty members and students. It confirmed that group work can induce behavioral changes among faculty members to improve HC. In the post-survey, this group work led the faculty members to caution themselves, other faculty members, and physicians to refrain from behaving in a manner that could trigger students’ unprofessional behavior. The group work promoted the realization that they were role models and provided an opportunity for the meta-awareness of their behavior.

The affinity diagram method made it easier for the participants to express their opinions, even if there were differences between the students and faculty members and among faculty members. This method can be conducted both face-to-face and online, using an online shared file. As the time required for the group work was short (around an hour), even busy medical professionals could participate. Some participants expressed hope that the group work would be repeated and performed by a wide range of medical professionals involved in various types of education programs for medical students and residents. Therefore, this group work is an easy-to-implement method to promote HC awareness and induce behavioral changes to improve HC for faculty members.

The results of Study 2 suggest that inappropriate educational environments and the statements, actions, and behaviors of senior students and educators may be HC that triggers students’ unprofessional behavior. If educators do not respond to learners’ unprofessional behavior, they implicitly condone the behavior and convey that it is not important or worth addressing [44]. However, managing students with unprofessional behavior requires faculty resources, time, and effort [44]. Therefore, it is important to create an educational culture and environment that prevents unprofessional behavior. The predisposing factors for unprofessional behavior include personal problems, interpersonal problems, external factors, and contextual factors [8]. Physicians’ and faculty members’ unprofessional behaviors were mentioned in the content analysis of the group work. Based on the findings, educators must be aware of their unprofessional behavior and be careful not to become negative role models. Along with strengthening the regulations and improving the educational environment, the development and introduction of FD is important for faculty members and physicians in addressing the HC identified in Study 2.

Furthermore, Study 2 shows that educators can make statements recommending unprofessional behavior. HC is transmitted through students’ observation of healthcare providers’ behavior, speech, tone, and attitudes toward their patients/environment and overall professional life [11]. Medical students often hear derogatory comments made by physicians to their patients [45–48] as they can understand the nuances and subtleties of communication that can make a statement derogatory [49]. Conversely, comments based on the past experiences of senior students, physicians, and faculty members may differ from these derogatory comments and arise in an attempt to have a good relationship with the student. Senior students, physicians, and faculty members may make comments based on their success, teaching experiences, and beliefs, which may have a positive educational effect on medical students. Notably, Hafferty states that stories, jokes, and personal anecdotes, whether told by faculty members or fellow students, can influence the educational process [9, 50].

This study explores medical students’ unprofessional behavior and the HC that triggers it in Japan, an Asian country. Therefore, cultural factors may have influenced the results. In an interview and questionnaire survey completed by Korean medical residents on doctors’ unprofessional behavior, substandard practice, violation of work ethics, dishonesty with patients, lack of respect for patients and colleagues, and misconduct in research were mentioned as doctors’ unprofessional behaviors [22, 24]. In a survey of Malaysian medical students’ unprofessional behavior, the three main problems observed were related to discipline, plagiarism and cheating, and sexual harassment [21]. These problems are similar to the behaviors of senior students, physicians, and faculty members, which is the HC identified in our study. The results of this study were obtained at a single center in Japan; hence, further reports are needed on whether similar results can be obtained in other Asian countries or whether these are unique Japanese characteristics.

Japan is teacher-centered and exam-led and encourages passive learning [27]. These characteristics are similar to those in the medical school education systems in other Asian countries [51]. Moreover, seniority is highly valued in most Japanese medical schools [25]. Therefore, statements from superiors are easily accepted by medical students. Appropriate guidance and role modeling by faculty members and senior physicians may encourage medical students to develop their skills. Murakami et al. reported that faculty enthusiasm could stimulate medical students and influence their choice and development of a career as a physician [25]. However, they suggested that negative role models may strongly reduce learner’s motivation. The application of these traditional Japanese educational practices in medical schools may cause problems associated with the hierarchical structure [25]. Educators should consider how their words and actions will be received. Tsai et al. report that in Confucian societies, which are common in Asia, senior leaders are influential, and developing an approach to HC can be desirable for such leaders [24]. The group work of this study can be implemented by medical school faculty members and students and may be effective in the context of other institutions and countries.

This study has five limitations. First, it was conducted in a medical school in Japan with a small number of participants. Therefore, both the impact of the group work on the participants and their comments are subject to cultural bias. Second, some faculty members were professors and the opinions of graduate students and residents were not collected. Third, the questionnaire items may not fully represent the emotions and attitudes of the participants because their validity and reliability have not been tested. Fourth, the survey results may be biased because of the low questionnaire collection rate. Fifth, the authors did not verify whether students’ unprofessional behaviors decreased as a long-term effect of the group work. Therefore, in future research, the number of participants should be increased based on the calculation of the required sample size, and a reliable questionnaire should be developed.

This group work can be conducted collaboratively with various educators and educational facilities, consolidating it into a comprehensive FD program. Its effectiveness can be assessed through continuous follow-ups with the participants and their respective facilities. In addition to monitoring behavioral changes in the participants, the final evaluation goal will be determining whether the incidence of unprofessional behavior among medical students has indeed been reduced.

## Conclusions

This study revealed that by engaging in group work in which opinions about HC are exchanged, faculty members and students can increase their awareness of the factors that trigger unprofessional behaviors. This study found that HC that triggers students’ unprofessional behavior includes the words and actions of the educator, organizational culture, and educational environment. Accordingly, educators should refrain from using words and actions that encourage unprofessional behavior, such as personal anecdotes, and that reduce students’ learning motivation. It is important to improve the educational environment for HC, as identified in this study, and approach faculty members and physicians through FD programs focusing on these issues. Furthermore, this group work can be repeated with different participants to constantly improve the learning environment.

### Electronic supplementary material

Below is the link to the electronic supplementary material.

Supplementary Material 1

Supplementary Material 2

## Data Availability

The datasets generated and/or analyzed during this study are available from the corresponding author upon reasonable request.

## Abbreviations

HC: Hidden curriculum
FD: Faculty development
