# Supplementary material for: Impact of group work on the hidden curriculum that induces students’ unprofessional behavior toward faculty
Source: BMC Med Educ. 2024 Jul 19;24:770. doi: 10.1186/s12909-024-05713-7 (PMC11264808; doi:10.1186/s12909-024-05713-7)
Supplement: Supplementary file 1 — Supplementary Material 1 [file 12909_2024_5713_MOESM1_ESM.docx]

**Supplementary Table 1. Questionnaire items in surveys regarding the group work.**

| **A. Survey immediately after the group work** | **Responses** |
| --- | --- |
| (A1) How useful was the group work? | 7-point Likert scale  1 (Not useful at all) — 4 (Neither) — 7 (Very useful) |
| (A2) How difficult was the group work? | 7 -point Likert scale  1 (Very easy) — 4 (Appropriate difficulty) — 7 (Very difficult) |
| (A3) How actively did you participate in the group work? | 7 -point Likert scale  1 (Very negative) — 4 (Neither) — 7 (Very positive) |
| (A4) How much new knowledge and insight did you gain? | 7 -point Likert scale  1 (Not gained at all) — 4 (Neither) — 7 (Greatly gained) |
| (A5) What was the good aspect of this group work? | Free comments |
| (A6) What was the negative aspect of this group work? | Free comments |
| (A7) Any additional comments or questions regarding the group work? | Free comments |
| **B. Survey six months after the group work** | **Responses** |
| (B1) To what extent did your daily educational behavior change as a result of the group work? | Seven-point Likert scale  1 (Not changed at all) — 4 (Neither) — 7 (Very much changed) |
| (B2) To what extent have you become more aware of other faculty members’ and physicians’ behaviors and environments that trigger students’ unprofessional behavior? | Seven-point Likert scale  1 (Not careful at all) — 4 (Neither) — 7 (Very careful) |
| (B3) How much would you like to participate in such professionalism training again? | Seven-point Likert scale  1 (Do not want to participate at all) — 4 (Neither) — 7 (Want to participate very much) |
| (B4) What did you implement after the group work to educate students about professionalism? | Free comments |
| (B5) What did you stop implementing for students’ professionalism education after the group work? | Free comments |
| (B6) Any additional comments or questions regarding the group work? | Free comments |
