# Supplementary material for: Impact of group work on the hidden curriculum that induces students’ unprofessional behavior toward faculty
Source: BMC Med Educ. 2024 Jul 19;24:770. doi: 10.1186/s12909-024-05713-7 (PMC11264808; doi:10.1186/s12909-024-05713-7)
Supplement: Supplementary file 2 — Supplementary Material 2 [file 12909_2024_5713_MOESM2_ESM.docx]

**Supplementary Table 2. List of themes created by the other groups using the affinity diagram method.**

| **Group** | **Theme of category** | **Selected theme** | **Cause** | **Management** |
| --- | --- | --- | --- | --- |
| 2 | 1. Personal anecdotes 2. Coercive 3. Arrogant 4. Lack of responsibility 5. Excessive appropriateness | - Personal anecdotes | - Generational disparities and shifting notions of time (when power harassment was commonplace in the past) - Excessive self-consciousness | - Faculty development held once a month - Notifying students that they do not find Personal anecdotes interesting - Students telling educators, ‘In this era, Personal anecdotes are uncool’. - Exploring other ways to display humanity and finding alternative means of captivation. - Presenting episodes that elevate student motivation. - Providing heart-stirring episodes involving patients. |
| 3 | 1. Harassment 2. Patient Relations 3. Misconduct 4. Clothing and appearance 5. Code of conduct 6. Behavior and dignity | - Harassment | - Historically rooted hierarchical contexts in the medical field - Insufficient mastery of appropriate teaching methods - Unconscious excitement since medical practice does not allow for mistakes | - Education program for doctors against harassment - Harassment training - Being aware of students’ presence during procedures - Having individuals available to support students who receive scolding after surgeries (follow-up for students as well) - Providing support systems for individuals who witness scolding or misconduct in clinical settings |
| 4 | 1. Ethics 2. Harassment 3. Study Attitude | - Ethics | - Diminished awareness of being observed by students - Faculty members have to extend their classes - Overemphasis on clinical practice - Downsides of online teaching | - Encouraging educators to be self-aware - Conducting reflection sessions for each specialty to share negative incidents - Reducing the bias in assignments given to students (creating a diversified curriculum) - Prioritizing students' well-being - Promoting information sharing between students and educators (providing accurate feedback) |
| 5 | 1. Inappropriate attitude as a medical professional 2. Grooming 3. Incorrect educational methods 4. Non-educational environment that reduces motivation | - Inappropriate attitude as a medical professional | - Displaying inappropriate attitudes as medical professionals - Making disrespectful remarks to patients - Lack of consciousness in using respectful language with patients - Assigning nicknames to patients - Displaying intimidating behavior toward patients and staff | - Educating residents and young doctors appropriately - Operating a well-organized team that fosters open communication - Re-evaluating work environments - Initiating reforms within the team - Considering students as future colleagues - Encouraging a shared sense of problem awareness among all faculty members - Considering the location, time, and situation before taking action |
| 6 | 1. Power harassment 2. Alcohol 3. Discourage learning. 4. Inappropriate comments or attitudes as a medical professional | - Undermining learning motivation | - Trying to show one’s abilities through such statements - Unconsciously recounting personal experiences - Lack of shared understanding to collectively aspire for excellence - Desire to assert superiority - Unable to spare time and effort for education - Demonstrating only minimum standards, without attempting higher goals | - Being aware of the potential to undermine learners’ motivation as an educator - Recognizing the possibility of making remarks that may diminish students’ motivation - Individually acknowledging locations and occasions where inappropriate statements are likely to occur |
